# Why does patients’ discharge delay after vertebral augmentation? A factor analysis of 1,442 patients

**DOI:** 10.3389/fsurg.2022.987500

**Published:** 2022-09-23

**Authors:** He Zhao, Zhengping Zhang, Yanjun Wang, Bing Qian, Xinhao Cao, Ming Yang, Yangjin Liu, Qinpeng Zhao

**Affiliations:** ^1^ Department of Emergency Medicine, Honghui Hospital, Xi’an Jiaotong University, Xi’an, China; ^2^ Department of Spine Surgery, Honghui Hospital, Xi’an Jiaotong University, Xi’an, China

**Keywords:** vertebral compression fracture, vertebral augmentation, vertebroplasty, delayed discharge, residual pain

## Abstract

**Objective:**

Vertebral augmentation techniques are widely used to treat osteoporotic vertebral compression fractures (OVCFs). Superior analgesic effects and shortened bed rest time means patients recover quickly, but prolonged unscheduled hospitalization can increase medical expenses and the risk of bed rest complications. The aim of this study was to investigate the reasons for prolonged hospitalization after vertebral augmentation surgery and to determine the relative risk factors.

**Methods:**

A single-center retrospective study was conducted to enroll patients with OVCFs and accepted vertebral augmentation surgery from January 2017 to December 2017. Clinical information was collected from the Hospital Information System (HIS). The criterion of delayed discharge was postoperative hospitalization more than 3 days. Telephone interviews and medical history evaluations were conducted to confirm the exact reason for retention. The risk factors were analyzed by multiple logistic regression.

**Results:**

Overall, 1,442 patients were included, and 191 (13.2%) stayed in the hospital for more than 3 days postoperatively. The reasons for delayed discharge were psychological factors (37.2%), residual pain (32.5%), cardiopulmonary complications (15.7%), nonspecific symptoms (8.4%), incision abnormalities (2.6%), thrombosis (2.1%), and postanesthesia reactions (1.6%). The multiple logistic model was significant; age (OR 1.028; 95% CI 1.009–1.046), preoperative stay (OR 1.192; 95% CI 1.095–1.298), operation type (OR 1.494; 95% CI 1.019–2.189), and the number of surgical segments (OR 2.238; 95% CI 1.512–3.312) showed statistical significance. In contrast, gender (*P* > 0.1) and chronic comorbidities (*P* > 0.1) were not predictors in this model.

**Conclusion:**

Overall, 13.2% of OVCF patients who underwent vertebral augmentation surgery were not discharged within 3 days postoperatively, and several predictors were found. Preoperative communication and comprehensive evaluations are calling for more attention; physicians should adopt an appropriate medical process to enhance rehabilitation in geriatric orthopedics.

## Introduction

Osteoporosis has become a global disease of the elderly that develops with age and is thought to be the underlying cause of osteoporosis fractures (OFs). Osteoporosis vertebral compression fractures (OVCFs) are an important component of OFs, as approximately 520,000 incidents occurred in the European Union in 2010 (1). Symptomatic OVCFs cause severe pain, lead to inferior quality of life, and are related to increased mortality risk (2). Vertebral augmentation, including percutaneous vertebroplasty (PVP) and percutaneous kyphoplasty (PKP), is commonly used to treat acute OVCFs. These methods require less operating time, are minimally invasive, and have higher cost-effectiveness compared with conservative treatments. Patients receiving PVP/PKP experienced pain relief and functional recovery. Reduction of hospitalization time can not only save medical resources and the financial burden of patients but also reduce bed rest time, which is one inducement of imbalanced bone turnover (3, 4). Research has shown that even short-term bed rest after trauma increased acute bone resorption, along with decreased muscle strength and aerobic capacity (5). Therefore, vertebral augmentation has a unique advantage in treating acute OVCFs in the elderly (6, 7).

The application of vertebral augmentation technology calls for the concept of rapid rehabilitation in geriatric orthopedics, including the removal of preoperative fear, surgical confidence, postoperative rehabilitation training, and functional recovery. However, we observed that some patients could not be discharged within the scheduled time and even undergo successful surgery, which may be caused by various factors. A randomized controlled trial reporting that 23% of acute OVCFs retained chronic low back pain after PVP (8) caused concern about residual symptoms. Meanwhile, severe cement leakages were reported, despite low complication morbidity (9). Furthermore, poor health conditions of the elderly increase the risk of acute onset of chronic diseases. All of the above-mentioned points out that prolonged bed rest will lead to more complications of being bedridden and a growing number of financial expenditures of patients (10).

Database searching found no relevant study on prolonged hospitalization or delayed discharge after PKP/PVP surgery. Therefore, the purpose of this study was to investigate the causes and predictors of delayed postoperative discharge to provide an informative clinical reference for the rehabilitation of patients with OVCFs.

## Materials and methods

We retrospectively reviewed patients who accepted PVP or PKP in our spine surgery department from January 1, 2017, to December 31, 2017, and all of the participants were in-patients. This study was performed in line with The Code of Ethics of the World Medical Association (Declaration of Helsinki) and was approved by the ethics committee of Honghui Hospital affiliated with Xi’an Jiaotong University.

### Inclusion and exclusion criteria

All patients had to meet the following inclusion criteria: (1) persistent back pain after slight exertion of energy or trauma and no evidence of relief; (2) clinical and imaging examinations, including x-ray, computed tomography, and magnetic resonance imaging presenting an OVCF related to the back pain; (3) osteoporosis diagnosis by dual-energy x-ray absorptiometry; and (4) complete information in the medical record system. The exclusion criteria are the following: (1) pathological fractures, including hemangioma and spinal metastasis; (2) chronic fractures, vertebrae osteonecrosis (like Kümmell disease), and intravertebral vacuum cleft in the vertebrae; (3) other coexisting traumas in addition to the spine (rib, limb, and sacrum fractures); and (4) severe comorbidities and other local or systematic disorders that may prolong the hospitalization.

To obtain the most consistent results according to the clinical situation, we did not limit the number of surgical segments or the age of participants. Preoperative examinations including lower limb arteriovenous ultrasound, blood routine, liver and kidney function, electrolyte, coagulation index, and infectious diseases were routinely performed. Patients with unstable comorbidities were consulted with relevant departments, and surgical treatment was performed only after excluding contraindications. All patients were informed of the treatment strategies by the physician, including operating procedures and prognosis.

### Surgical procedures

PVP or PKP was chosen according to the specific fracture form and economic condition. PVP combined with the free-hand reduction was considered when the compression degrees of vertebrae anterior column were less than 30%; PKP and free-hand reduction were preferred in patients with greater than 30% compression. Patients who required a PVP due to poor economic conditions were informed about the risk and signed a consent form.

All procedures were conducted with standard procedures by senior spinal surgeons. Antibiotics were used intravenously 1 h prior to the procedure. The free-hand reduction was performed in a prone position, and a moderate restoration under x-ray was acceptable. Most of the patients were treated under infiltration anesthesia with 1% lidocaine, while a few others were treated with general anesthesia (in consideration of strong fear of surgery). One or two trocars (KINETIC, China) were inserted into the pedicles of the object vertebrae under the surveillance of a C-arm x-ray (GE, American). The needles were inserted at the 3 or 9-o’clock position of pedicles with a specific inclination to approach the anterior third of the vertebrae body on anteroposterior and lateral radiographs. The vertebrae were then expanded by a balloon in PKP procedures. Pasta-like polymethylmethacrylate (PMMA, KINETIC, China) was injected until the cement approaching the posterior wall of the vertebrae or cement leakage was observed.

Patients remained in bed after surgery, and x-ray examination was undertaken within 12 h to ensure the cement location was good. Patients were advised to walk moderately with a plastic thoracolumbosacral orthosis (Hengshui Qianzhong Medical Equipment Co. Ltd., China) routinely on the second day after surgery. A 3-month brace stabilization was generally recommended.

### Demographic data

To analyze the factors of delayed discharge after PKP/PVP, relevant clinical information covering gender, age, prehospital time, pre- and postoperative stays, preoperative bone mineral density (BMD), preoperative VAS, operation type, the number of surgical segments, and complete admitting/discharge diagnosis was obtained from the Hospital Information System (HIS). The third-day postoperatively VAS score was recorded to represent the pain relief at discharge. Diagnosis including cardiopulmonary diseases, hypertension, liver and kidney dysfunction, and diabetes was recorded as chronic comorbidities. Telephone interviews and medical history research were conducted to confirm the main reason for not being discharged on time.

### Clinical outcomes

We used the following discharge criteria: (1) successful operation, no severe surgical complications such as cement embolism (in pulmonary arteries or cerebrovascular vessels) and intraspinal leakage (compressing the spinal cord or nerve root), which usually leads to urgent interventional thrombectomy or spinal decompression surgery; (2) visual analog scale (VAS) score decreased to below 4 (at most slight pain) (11–13); and (3) stable life signs, no acute comorbidities.

Subject to the requirements of local medicare policy and our hospital's clinical pathway, patients after vertebral augmentation surgery should be discharged within 3 days if they meet the above standard. Therefore, in this study, delayed discharge was considered to be postoperative hospitalization time over 3 days.

### Statistical analyses

All statistical analyses were performed with Statistical Packages for Social Sciences V21.0 for Windows (SPSS Inc. Chicago, IL, USA). To describe the basic characteristics of the patients, quantitative variables were reported by means and standard deviations, while counts and percentages were recorded for qualitative variables. Chi-square tests and *t*-test/non-parametric tests were performed for univariate analyses, and *P* values <0.1 were considered significant temporarily. The Box–Tidwell test was used to verify whether a linear relationship existed between continuous independent variables and logit conversion values of dependent variables. Then, binary logistic analysis was performed to identify the predictors and odds ratios for delayed discharge, and *P* values <0.05 were considered significant.

## Results

**Figure 1:**
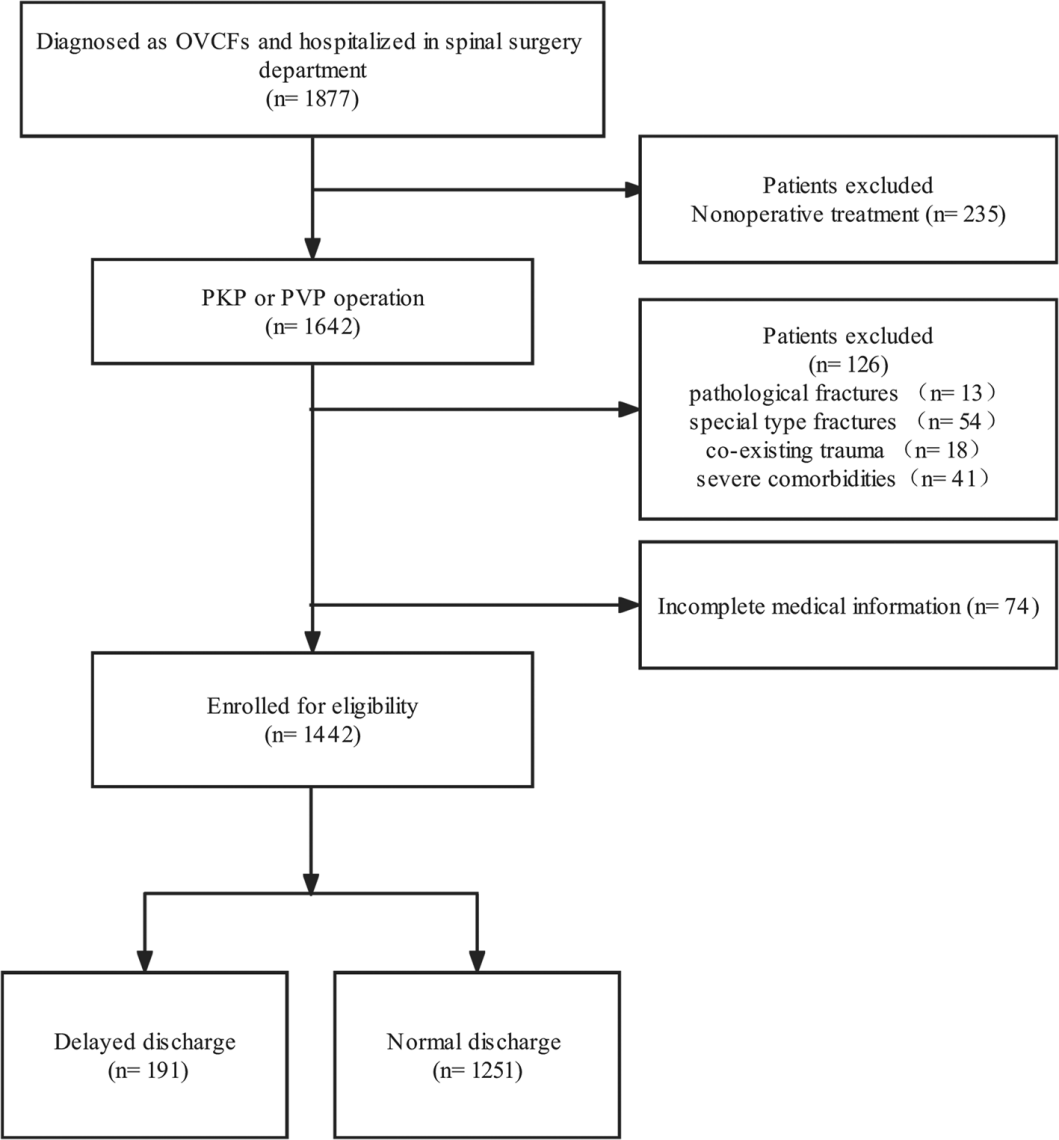
Study flowchart.

After filtering 1,877 patients who underwent PVP or PKP from January 1, 2017, to December 31, 2017, 1,442 patients (295 males and 1,147 females) with 1,549 treated vertebrae (1–4) were included in our study (Figure 1). The mean age of all patients was 71.95 ± 8.79 years (range 47–95). At a mean of 2.09 ± 1.33 days after injury, all patients came to the hospital and stayed for 4.01 ± 2.55 days in total, including 1.93 ± 1.58 days for preoperation and 2.06 ± 1.63 days for postoperation. The mean preoperative BMD and pre- and third-day postoperative VAS are shown in Table 1. Overall, 191 (13.2%) patients (mean age 73.58 ± 8.28) stayed in the hospital for more than 3 days after the surgery. The length of stay after the surgery was 5.09 ± 2.21 days (range 4–25), while the people discharged in 3 days had a shorter length of 1.59 ± 0.83 days (range 0–3) (Table 1).

**Table 1:** Clinical information and characteristics of patients.

Variables	Delayed discharge	Normal discharge	Overall
Patients with surgery (*n*)	191 (13.2%)	1,251 (86.8%)	1,442 (100%)
Age (y)	73.58 ± 8.28	71.70 ± 8.84	71.95 ± 8.79
Gender (m/f)	49/142	246/1,005	295/1,147
Prehospital time (d)	2.19 ± 1.45	2.08 ± 1.32	2.09 ± 1.33
Preoperative time (d)	2.42 ± 1.45	1.86 ± 1.58	1.93 ± 1.58
Preoperative VAS	7.73 ± 0.97	7.52 ± 0.91	7.54 ± 0.92
Preoperative BMD (*T*-score)	−3.71 ± 0.59	−3.66 ± 0.69	−3.67 ± 0.68
Postoperative stay (d)	5.09 ± 2.22	1.59 ± 0.83	2.06 ± 1.63
Third-day postoperative VAS	2.90 ± 2.42	1.60 ± 1.03	1.77 ± 1.38
Chronic comorbidities (y/n)	100/91	615/636	715/727
Operation type (K/V)	153/38	903/348	1056/386
Number of segments (*n*)	1.17 ± 0.47	1.06 ± 0.26	1.07 ± 0.30

Data were mean ± SD or *N* (%); *n* = number, y = years, m/f = male/female, K/V = PKP/PVP, d = days, y/n = yes/no.

After referring to the case history, we recorded chronic comorbidities including hypertension, diabetes mellitus, coronary heart disease, arrhythmia, cerebral infarction, and chronic obstructive pulmonary disease. Overall, 715 (49.6%) patients suffered one or more chronic comorbidities before hospitalization, and the rest of the patients simply had osteoporosis except for other local diseases, which were not recognized as comorbidities (Table 1).

**Figure 2:**
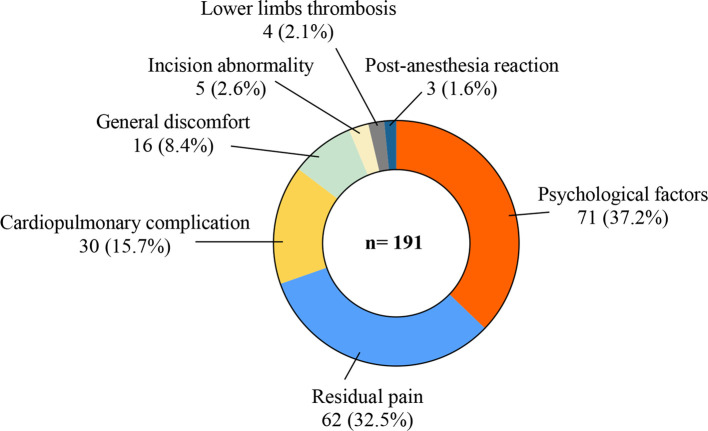
Reasons for delayed discharge.

We carried out phone interviews combined with medical history research in HIS, summarized the main reasons for the delay, and sorted these in Figure 2. In total, 71 (37.2%) of 191 patients met the discharge criteria but required extra treatment, mainly concerning their physical condition relating to trauma and surgery. We regarded these as psychological factors and gave them conservative treatment and nutrition support therapy until all these patients were discharged to communities or rehabilitation facilities. Sixty-two (32.5%) patients complained of residual pain (VAS value ≥4, range 4–9) from the former location or elsewhere after the surgery. Conservative analgesia therapies like oral NSAIDs or diclofenac lidocaine intramuscular injection were performed daily in these situations, and all these patients were relieved to varying degrees and then finally discharged. Cardiopulmonary complications, including acute heart failure, atrial fibrillation, and pneumonia, were the third reason that caused 30 (15.7%) patients to prolong their postoperative stay. They got emergency treatments and were transferred to specific departments with medical consultations. In addition, 16 (8.4%) experienced general discomfort, covering fever, stomachache, and headache and gradually recovered after symptomatic treatments and observations. Five (2.6%) incision abnormalities, four (2.1%) lower limb thromboses, and three (1.6%) postanesthesia reactions were recorded. No patient sustained severe cement leakage that needed reoperation including interventional therapy or spinal canal decompression.

We put gender, age, prehospital time, preoperative BMD, pre- and third-day postoperative VAS, preoperative stays, operation type, number of surgical segments, and chronic comorbidities into univariate analyses after all quantitative variables were proven to be nonnormally distributed. Gender, operation type, and chronic comorbidities were transformed into categorical data. As the results show in Table 2, age (*P* < 0.05), preoperative time (*P* < 0.001), preoperative VAS (*P* < 0.05), third-day postoperative VAS (*P* < 0.001), operation type (*P* < 0.05), and the number of surgical segments (*P* < 0.001) showed significance to delayed discharge, while prehospital time (*P* = 0.484), preoperative BMD (*P* = 0.396), and chronic comorbidities (*P* > 0.1) were not significant between the two groups. Gender (*P* = 0.056) approached statistical significance and was included in the multivariate analysis. A binary logistic analysis was performed to investigate the predictors of delayed discharge. Third-day postoperative VAS was excluded for direct relation to delayed discharge. The Box–Tidwell test showed a linear relationship between continuous independent variables and logit conversion values of dependent variables. Collinearity diagnostics showed negative results between the independent variables. Overall, the logistic model was significant (*χ*^2^ = 56.796, *P* < 0.001). Age (OR 1.028; 95% CI 1.009–1.046), preoperative time (OR 1.181; 95% CI 1.084–1.288), preoperative VAS (OR 1.271; 95% CI 1.070–1.510), operation type (OR 1.501; 95% CI 1.023–2.201), and the number of surgical segments (OR 2.231; 95% CI 1.503–3.310) showed statistical significance. Gender (*P* = 0.103) was not a predictor of delayed discharge of patients after PVP/PKP (Table 3).

**Table 2:** Univariate analysis of factors for delayed discharge.

Variables	*χ*^2^/*Z*	P* value
Gender	3.654	0.056
Chronic comorbidities	0.677	0.411
Operation type	5.305	0.021
Age	−2.574	0.010
Prehospital time	−0.700	0.484
Preoperative time	−6.089	<0.001
Preoperative VAS	−2.525	0.012
Third day postoperative VAS	−6.036	<0.001
Preoperative BMD	−0.849	0.396
Number of segments	−4.561	<0.001

VAS, visual analog scale.

* Statistics were analyzed using the chi-square test and Mann–Whitney *U* test.

**Table 3:** Multivariate logistic analysis for delayed discharge.

Variety of factors	*B*	S.E.	Wald *χ*^2^	OR	CI 95%	*P* value
Gender	0.303	0.186	2.652	1.353	0.940	1.948	0.103
Age	0.027	0.009	8.898	1.028	1.009	1.046	**0.003**
Preoperative time	0.167	0.044	14.292	1.181	1.084	1.288	**<0.001**
Preoperative VAS	0.240	0.088	7.451	1.271	1.070	1.510	**0.006**
Operation type	0.406	0.195	4.322	1.501	1.023	2.201	**0.038**
Number of segments	0.802	0.201	15.873	2.231	1.503	3.310	**<0.001**

Gender and operation type were transferred into categorical data; B, partial regression coefficient; S.E., standard error; OR, odds ratio; CI, confidence interval; *P* values in bold were statistically significant.

## Discussion

OVCFs in patients with osteoporosis involve severe pain episodes. The efficacy of conservative bed rest treatment is still uncertain but can cause complications such as muscle weakness, atelectasis, thrombosis, and pressure ulcers (10). Previous research has reported that bone resorption increases from the second day of bed rest (3), implying the disadvantages of immobilization. Vertebral augmentation is widely used to restore OVCF patients more quickly; meanwhile, the procedure being performed as ambulatory surgery is growing (14), as minimally invasive and rapid surgical intervention measures are effective and acceptable for elderly patients with OVCFs. Delayed discharge is an important quality monitor in ambulatory surgeries, and prolonged postoperative hospitalization may be related to poor quality of care and patients’ low acceptance of ambulatory surgery, which may affect its superior cost-effectiveness (15).

To our knowledge, two high-quality studies about PVP surgery were published in 2009, querying the effectiveness of PVP and causing considerable controversy (16, 17). However, further studies have been conducted in subsequent clinical trials with strictly formulated inclusion criteria; PVP achieved more significant pain relief and vertebral height recovery than sham surgery (6). Nevertheless, current studies have shown similar analgesic effects of PVP and PKP (18). In this study, we have to note that all patients enrolled were in-patients because PVP/PKP were not carried out as ambulatory surgeries in our hospital during that time. Even so, our clinical pathway for the PKP/PVP operation required unified surgical and discharge standards, and patients with permitted situations were advised to discharge within 3 days after the surgery. Therefore, we can still obtain meaningful results by using this discharge indicator and providing advice for clinical work.

This study showed postoperative information about in-patients after vertebral augmentation surgery. Under the unified discharge standards, 191 (13.2%) patients stayed in the hospital longer than 3 days postoperatively, which was considered delayed discharge. All of the above patients got relevant treatments and reassessment and were finally discharged in a few days (range 4–25 days postoperatively). According to telephone interviews and medical history analyses, the reasons for delayed discharge related to incidence were psychological factors (37.2%), residual pain (32.5%), cardiopulmonary complications (15.7%), general discomfort (8.4%), incision abnormalities (2.6%), thrombosis (2.1%), and postanesthesia reactions (1.6%) (Figure 2). To further analyze the factors influencing delayed discharge, age, gender, prehospital time, pre- and third-day postoperative VAS, preoperative BMD, preoperative time, operation type, the number of surgical segments, and chronic comorbidities were included in univariate and multifactor analyses.

As we present in Table 3, age (OR 1.028; 95% CI 1.009–1.046), preoperative time (OR 1.181; 95% CI 1.084–1.288), preoperative VAS (OR 1.271; 95% CI 1.070–1.510), operation type (OR 1.501; 95% CI 1.023–2.201), and number of surgical segments (OR 2.231; 95% CI 1.503–3.310) were independent risk factors for delayed discharge after vertebral augmentation surgery in in-patients. The results indicated that, with each additional year of age, each more VAS point preoperatively, each additional day of preoperative hospitalization, every additional surgical segment and PKP compared with PVP, the risk of delayed discharge increased by 2.8, 18.1, 27.1, 50.1 and 123.1%, respectively. However, there were no significant associations between delayed discharge and gender, preoperative BMD, prehospital time, or chronic comorbidities. All of the factors from the logistic analysis will be discussed in the following sections.

### Psychological factors

As previously mentioned, psychological factors were the most common reason for delayed discharge in this study, accounting for 37.2%. All of these patients had successful surgery and significant pain relief (VAS < 4) and met the discharge criteria. However, they rejected the discharge advice and asked for further conservative treatment in the hospital. Patients tend to like more comprehensive therapy when a fracture incident led to surgery, even if the pain got prominent relief. Mental disorders were excluded, and the feasibility of discharge was told to the patients and agents. Conservative treatment such as antiosteoporosis medication (calcitonin or intravenous bisphosphonates) and functional rehabilitation exercises was conducted. A retrospective study investigated the disposition of hospitalized patients after PVP. Approximately one-half of the patients (44%) living at home before surgery were discharged to rehabilitation facilities after surgery (19). Other areas of research, such as day-surgery laparoscopic cholecystectomy, have reported some factors responsible for delayed discharge, including psychosocial factors (20). On the one hand, patients usually believe that they should get more professional care than unsupervised rehabilitation at home. On the other hand, elderly age and comorbidities may burden them. It is the clinician’s responsibility to understand the patient's perception. Another study from Sweden (21) surveyed patients after ambulatory surgery and reported that psychological preparation, knowledge of recovery, rehabilitation assistance, and a sense of security were required when patients returned home. Meanwhile, poor preoperative conversations may result in inadequate preparation and excessive concerns; less home assistance also leads to rejection for returning home early, which indeed requires nursing strategies and rehabilitation centers. In our opinion, adequate psychological preparation is a prerequisite for elderly OVCF patients facing surgery or discharge. Patients need to know more about the experience of rapid recovery, therapeutic schedule, postoperative matters, and long-term rehabilitation; perioperative care of nurses was also indispensable.

### Residual pain

Back pain is the leading symptom of OVCFs, but it could also come from adjacent soft tissue injuries. Thus, it is rational that pain relief is a subjective measure of surgical efficacy. In this study, patients with other injuries (such as distant fractures) were excluded to reduce bias, but inconspicuous injuries adjacent to the vertebrae were hard to detect. Sixty-two patients in the study complained of medium-to-severe pain (VAS value ≥4, range 4–9) after surgery, which was considered residual pain. Analgesic therapy was used after confirming no missing vertebral fractures, and all these patients got different levels of relief when finally discharged.

From Table 3, we found that preoperative VAS (OR 1.271), the number of surgical segments (OR 2.231), operation type (OR 1.501), and preoperative time (OR 1.181) were independent risk factors that led to prolonged discharge, which might be together associated with residual pain. Generally, multiple fractures are likely combined with greater energy of trauma, thus resulting in enduring pain and higher preoperative VAS. Ten of 62 residual pain patients during follow-up claimed that they got great relief in thoracolumbar but felt significant pain in the posterior superior iliac. We supposed that the elderly with weak muscle tend to get extra injury in places other than the spine, especially in the posterior superior iliac for an accidental tumble, which needs further research. Yan et al. (22) believed that OVCF combined with thoracolumbar fascia injury was related to residual back pain after PVP, and the surgery always resolved spinal disorders but usually ignored peripheral soft tissue damage (23).

During the procedure, the leakage of bone cement around the vertebral body can also cause postoperative back pain (24). Although there were no spinal cord or nerve root compressions by cement, there was still a possibility of back pain derived from intervertebral disc leakage (24). We recorded no severe cement leakage incident, but leakages surrounding the vertebrae happened occasionally. It was suggested that the operation be standardized to avoid the leakage of bone cement and the damage to the transverse process and intervertebral joints.

Moreover, nonunion of OVCF (also called Kümmell disease) will cause long-term pain that is difficult to relieve. Ischemic necrosis and exudation formed in the nonunion vertebral body, which are not conducive to adequate fixation of bone cement, made the efficiency uncertain (25). We have excluded all of the Kümmell diseases and chronic fractures, and no osteonecrosis was observed after surgery.

According to the mechanism of vertebral augmentation surgery, the volume of bone cement filling is closely related to pain relief. Some studies have shown that sufficient cement filling helps stabilize the vertebral body and relieve pain (26, 27). However, a classic study showed that 15% of the volume of the vertebral body could be filled to achieve effective safe balance (28). It is noteworthy that multiple factor analysis found that operation type (OR 1.501) was a predictor, indicating that PKP has a higher risk for delayed discharge than PVP. In this study, PVP usually performed on relatively slight compression vertebrae may be a reason. However, we suppose that cement filling in PVP could be more diffuse than a mass usually in PKP that may have a better analgesia effect, although previous studies suggested there was no significant difference in pain relief between PVP and PKP (18, 29).

In general, many issues influence residual pain after surgery, and no consensus has been reached. It should be noted that a comprehensive and accurate diagnosis before surgery plays a crucial role. Assessment of curative effects should be emphasized when accompanying adjacent injury. Furthermore, significant degeneration in the elderly also reminds us to identify the pain source accurately.

### Age and cardiopulmonary complications

The mean age of the patients in this study was 71.95 ± 8.79 years, among which the delayed discharge population was 73.58 ± 8.28 (Table 1). Elderly patients are often admitted with various chronic diseases, with a risk of acute complications under trauma and surgical stress conditions. We have observed arrhythmias, atrial fibrillation, acute heart failure, acute hypertension, and acute exacerbation of chronic obstructive pulmonary diseases. All 30 patients with complications received in-hospital consultation with treatment suggestions, and serious cases were transferred to a specialized department. It is worth discussing that, although all of these patients were admitted with chronic diseases, the chronic comorbidity indicators were not predictors of delayed discharge after multifactor analysis (Table 3). We hypothesized that this was due to the high average age; there was an approximate rate of chronic comorbidities existing between the normal group and the delayed group (Table 1). The details of existing chronic comorbidities were yes or no, so it was difficult to distinguish the severity of specific diseases solely by diagnosis information in the medical records. In addition, high-risk patients were excluded from the surgical plan, resulting in selection bias. The occurrence of acute complications may be accidental. For this reason, clinicians need to strengthen the comprehensive evaluation during the perioperative period to reduce accidents.

### Other events

Sixteen patients developed nonspecific symptoms of discomfort and complained of fever, headache, and stomachache, and general treatment was effective. Five patients reported incision pain but denied obvious deep structure pain while moving. We observed slight redness and swelling in the incisions, no fluid exudation, and neither fever nor abnormal laboratory results. As we know, vertebral augmentation has a rare incidence of infection, but it can still be a formidable and life-threatening complication. Surgery should be avoided for patients with infectious tendencies, and preventive antibiotic therapy should be conducted for those with low immune function in the perioperation period (30). Four thromboses might have been associated with bed rest under stress. Patients suffering from OVCF usually seek doctors after days of bed rest. Continuous immobilization and prolonged pressure on the limbs result in venous stasis, posing a risk of thrombosis (10). For the anesthesia methods, we usually choose infiltration anesthesia due to its safety and convenience; a small portion of patients underwent general anesthesia in consideration of pain stimulation. There were fewer cases of prolonged hospitalization due to postanesthesia reactions, which depended on a detailed preanesthesia evaluation.

### Limitations

There were several limitations in the present study. First, only a few indicators of medical information were collected in this study. Thus, part of the results in the regression analysis seemed to be nondistinct, such as preoperative stay; it has not been completely explained how the preoperative extension prolonged the length of postoperation. Second, details of cement leakage in all 1,442 patients were not reported, although leakage surrounding the vertebrae can also cause postoperative residual pain. Further research requires improved clinical data for more details.

## Conclusion

Overall, 13.2% of patients in this study who underwent vertebral augmentation surgery were not discharged within 3 days after surgery. The most common causes are psychological factors, residual pain, and cardiopulmonary complications. Multifactor analysis revealed that age, number of surgical segments, operation type, and preoperative stay were the main factors related to delayed discharge. Preoperative communication and comprehensive evaluations are calling for more attention, and physicians should adopt an appropriate medical process to enhance rehabilitation in geriatric orthopedics.

## Data Availability

The raw data supporting the conclusions of this article will be made available by the authors, without undue reservation.

## References

[1] Hernlund E Svedbom A Ivergård M Compston J Cooper C Stenmark J Osteoporosis in the European union: medical management, epidemiology and economic burden. A report prepared in collaboration with the international osteoporosis foundation (IOF) and the European federation of pharmaceutical industry associations (EFPIA). Arch Osteoporos. (2013) 8(1):136. 10.1007/s11657-013-0136-1. 24113837 PMC3880487

[2] Bliuc D Nguyen ND Milch VE Nguyen TV Eisman JA Center JR. Mortality risk associated with low-trauma osteoporotic fracture and subsequent fracture in men and women. J Am Med Assoc. (2009) 301(5):513–21. 10.1001/jama.2009.50. 19190316

[3] Baecker N Tomic A Mika C Gotzmann A Platen P Gerzer R Bone resorption is induced on the second day of bed rest: results of a controlled crossover trial. J Appl Physiol. (2003) 95(3):977–82. 10.1152/japplphysiol.00264.2003. 12909597

[4] Kortebein P Symons TB Ferrando A Paddon-Jones D Ronsen O Protas E Functional impact of 10 days of bed rest in healthy older adults. J Gerontol A Biol Sci Med Sci. (2008) 63(10):1076–81. 10.1093/gerona/63.10.1076. 18948558

[5] Rittweger J Felsenberg D. Recovery of muscle atrophy and bone loss from 90 days bed rest: results from a one-year follow-up. Bone. (2009) 44(2):214–24. 10.1016/j.bone.2008.10.044 19022418

[6] Clark W Bird P Gonski P Diamond TH Smerdely P McNeil HP Safety and efficacy of vertebroplasty for acute painful osteoporotic fractures (VAPOUR): a multicentre, randomised, double-blind, placebo-controlled trial. Lancet. (2016) 388(10052):1408–16. 10.1016/S0140-6736(16)31341-1 27544377

[7] Yang EZ Xu JG Huang GZ Xiao WZ Liu XK Zeng BF Percutaneous vertebroplasty versus conservative treatment in aged patients with acute osteoporotic vertebral compression fractures: a prospective randomized controlled clinical study. Spine. (2016) 41(8):653–60. 10.1097/BRS.0000000000001298. 26630417

[8] Peris P Blasco J Carrasco JL Martinez-Ferrer A Macho J San Roman L Risk factors for the development of chronic back pain after percutaneous vertebroplasty versus conservative treatment. Calcif Tissue Int. (2015) 96(2):89–96. 10.1007/s00223-014-9940-x. 25492165

[9] Saracen A Kotwica Z. Complications of percutaneous vertebroplasty: an analysis of 1100 procedures performed in 616 patients. Medicine. (2016) 95(24):e3850. 10.1097/MD.0000000000003850. 27310966 PMC4998452

[10] Brower RG. Consequences of bed rest. Crit Care Med. (2009) 37(10 Suppl):S422–8. 10.1097/CCM.0b013e3181b6e30a. 20046130

[11] Yang JS Liu JJ Chu L Li J Chen C Chen H Causes of residual back pain at early stage after percutaneous vertebroplasty: a retrospective analysis of 1,316 cases. Pain Physician. (2019) 22(5):E495–503. https://www.painphysicianjournal.com/current/pdf?article=NjUxOQ%3D%3Djournal=122 31561662

[12] Xu JJ Tang XT Yang J Wang YH Zhu DC Wu YS The effect of medial branch block on postoperative residual pain relieve after percutaneous kyphoplasty: a randomized controlled trial with 12-month follow-up. Pain Physician. (2021) 24(7):E1059–E66. https://www.painphysicianjournal.com/current/pdf?article=NzM0OQ%3D%3Djournal=139 34704715

[13] Price DD McGrath PA Rafii A Buckingham B. The validation of visual analogue scales as ratio scale measures for chronic and experimental pain. Pain. (1983) 17(1):45–56. 10.1016/0304-3959(83)90126-4 6226917

[14] Goz V Koehler SM Egorova NN Moskowitz AJ Guillerme SA Hecht AC Kyphoplasty and vertebroplasty: trends in use in ambulatory and inpatient settings. Spine J. (2011) 11(8):737–44. 10.1016/j.spinee.2011.07.002 21862416

[15] Shirakami G Teratani Y Namba T Hirakata H Tazuke-Nishimura M Fukuda K. Delayed discharge and acceptability of ambulatory surgery in adult outpatients receiving general anesthesia. J Anesth. (2005) 19(2):93–101. 10.1007/s00540-004-0297-6 15875124

[16] Kallmes DF Comstock BA Heagerty PJ Turner JA Wilson DJ Diamond TH A randomized trial of vertebroplasty for osteoporotic spinal fractures. N Engl J Med. (2009) 361(6):569–79. 10.1056/NEJMoa0900563 19657122 PMC2930487

[17] Buchbinder R Osborne RH Ebeling PR Wark JD Mitchell P Wriedt C A randomized trial of vertebroplasty for painful osteoporotic vertebral fractures. N Engl J Med. (2009) 361(6):557–68. 10.1056/NEJMoa0900429 19657121

[18] Marcia S Muto M Hirsch JA Chandra RV Carter N Crivelli P What is the role of vertebral augmentation for osteoporotic fractures? A review of the recent literature. Neuroradiology. (2018) 60(8):777–83. 10.1007/s00234-018-2042-0 29947942

[19] Harvey RE Kallmes DF. Discharge disposition following vertebroplasty. Am J Neuroradiol. (2011) 32(9):1614–6. 10.3174/ajnr.A2580. 21816917 PMC7965381

[20] Cao J Liu B Li X Leng J Meng X Pan Y Analysis of delayed discharge after day-surgery laparoscopic cholecystectomy. Int J Surg. (2017) 40:33–7. 10.1016/j.ijsu.2017.02.055 28235668

[21] Berg K Arestedt K Kjellgren K. Postoperative recovery from the perspective of day surgery patients: a phenomenographic study. Int J Nurs Stud. (2013) 50(12):1630–8. 10.1016/j.ijnurstu.2013.05.002 23726224

[22] Yan Y Xu R Zou T. Is thoracolumbar fascia injury the cause of residual back pain after percutaneous vertebroplasty? A prospective cohort study. Osteoporos Int. (2015) 26(3):1119–24. 10.1007/s00198-014-2972-2 25510580

[23] Gibson JE Pilgram TK Gilula LA. Response of nonmidline pain to percutaneous vertebroplasty. Am J Roentgenol. (2006) 187(4):869–72. 10.2214/AJR.05.0084. 16985127

[24] Wang CH Ma JZ Zhang CC Nie L. Comparison of high-viscosity cement vertebroplasty and balloon kyphoplasty for the treatment of osteoporotic vertebral compression fractures. Pain Physician. (2015) 18(2):E187–94. https://www.painphysicianjournal.com/current/pdf?article=MjI3OA%3D%3D/journal=87 25794218

[25] Peh WC Gelbart MS Gilula LA Peck DD. Percutaneous vertebroplasty: treatment of painful vertebral compression fractures with intraosseous vacuum phenomena. Am J Roentgenol. (2003) 180(5):1411–7. 10.2214/ajr.180.5.1801411. 12704060

[26] Fu Z Hu X Wu Y Zhou Z. Is there a dose–response relationship of cement volume with cement leakage and pain relief after vertebroplasty? Dose Response. (2016) 14(4):1559325816682867. 10.1177/1559325816682867 28182178 PMC5283639

[27] He SC Teng GJ Deng G Fang W Guo JH Zhu GY Repeat vertebroplasty for unrelieved pain at previously treated vertebral levels with osteoporotic vertebral compression fractures. Spine. (2008) 33(6):640–7. 10.1097/BRS.0b013e318166955f. 18344858

[28] Liebschner MA Rosenberg WS Keaveny TM. Effects of bone cement volume and distribution on vertebral stiffness after vertebroplasty. Spine. (2001) 26(14):1547–54. 10.1097/00007632-200107150-00009. 11462084

[29] Zhao S Xu CY Zhu AR Ye L Lv LL Chen L Comparison of the efficacy and safety of 3 treatments for patients with osteoporotic vertebral compression fractures: a network meta-analysis. Medicine. (2017) 96(26):e7328. 10.1097/MD.0000000000007328. 28658144 PMC5500066

[30] Abdelrahman H Siam AE Shawky A Ezzati A Boehm H. Infection after vertebroplasty or kyphoplasty. A series of nine cases and review of literature. Spine J. (2013) 13(12):1809–17. 10.1016/j.spinee.2013.05.053 23880354

